# Induction of the Unfolded Protein Response at High Temperature in *Saccharomyces cerevisiae*

**DOI:** 10.3390/ijms23031669

**Published:** 2022-01-31

**Authors:** Tatsuya Hata, Yuki Ishiwata-Kimata, Yukio Kimata

**Affiliations:** Division of Biological Science, Graduate School of Science and Technology, Nara Institute of Science and Technology, 8916-5 Takayama, Ikoma, Nara 630-0192, Japan; naist11-hata@yahoo.co.jp (T.H.); naist11-yuki@yahoo.co.jp (Y.I.-K.)

**Keywords:** endoplasmic reticulum, yeast, mRNA solicing, PCR

## Abstract

Ire1 is an endoplasmic reticulum (ER)-located endoribonuclease that is activated in response to ER stress. In yeast *Saccharomyces cerevisiae* cells, Ire1 promotes *HAC1-*mRNA splicing to remove the intron sequence from the *HAC1*u mRNA (“u” stands for “uninduced”). The resulting mRNA, which is named *HAC1*i mRNA (“i” stands for “induced”), is then translated into a transcription factor that is involved in the unfolded protein response (UPR). In this study, we designed an oligonucleotide primer that specifically hybridizes to the exon-joint site of the *HAC1*i cDNA. This primer allowed us to perform real-time reverse transcription-PCR to quantify *HAC1*i mRNA abundance with high sensitivity. Using this method, we detected a minor induction of *HAC1*-mRNA splicing in yeast cells cultured at their maximum growth temperature of 39 °C. Based on our analyses of *IRE1*-gene mutant strains, we propose that when yeast cells are cultured at or near their maximum growth temperature, protein folding in the ER is disturbed, leading to a minor UPR induction that supports cellular growth.

## 1. Introduction

The endoplasmic reticulum (ER) is the cellular compartment wherein secretory and membrane proteins are folded. In addition, membrane–lipid components are mainly synthesized on the ER. Impairment of or insufficiency of these ER functions is called ER stress. In response to ER stress, eukaryotic cells alter their gene expression profile. This protective response is known as the unfolded protein response (UPR) and is at least partly controlled by the ER-located type-I transmembrane protein Ire1 [1,2].

The mechanistic features of UPR were initially uncovered through frontier studies using the model organism *Saccharomyces cerevisiae* (hereafter simply called yeast) [3]. The luminal domain of Ire1 directly senses unfolded proteins that accumulate in the ER, leading to activation of Ire1 as an endoribonuclease [4,5,6]. Ire1 can also be activated by a membrane–lipid-related abnormality, namely, lipid bilayer stress (LBS), for which its transmembrane domain, but not its luminal domain, acts as the stress sensory module [7,8]. In other words, the accumulation of unfolded proteins in the ER and LBS are distinct types of ER stress-inducing stimuli that are both detected by Ire1, but in different ways.

In yeast cells, Ire1 targets *HAC1* gene transcripts, which are called *HAC1*u mRNA (“u” stands for “uninduced”). When activated, Ire1 promotes the splicing of *HAC1*u mRNA to yield *HAC1*i mRNA (“i” stands for “induced”). While *HAC1*u mRNA is translated ineffectively, *HAC1*i mRNA is translated into the transcription factor Hac1, which induces a large number of genes for UPR [9,10].

The mechanistic features of Ire1 activity have mainly been studied through the artificial induction of strong ER stress. Because protein folding in the ER is frequently accompanied with cysteine disulfide-bond formation, the thiol reductant dithiothreitol (DTT) induces strong ER stress. Beyond its mechanistic functioning, it remains unclear as to which conditions in nature activate Ire1 and induce UPR. Since the proximal target of yeast Ire1 is *HAC1*u mRNA, we anticipate that the activity of Ire1 in yeast cells can be sharply monitored by measuring the splicing of the *HAC1*u mRNA (the *HAC1*-mRNA splicing). However, as described later, *HAC1*-mRNA splicing is hardly detected by conventional techniques when its level is weak.

Unlike well-known ER-stress agents such as DTT, other stimuli may provoke *HAC1*-mRNA splicing only weakly. Therefore, we developed a method to quantitatively monitor the *HAC1*-mRNA splicing, even when it occurs at a low level. This method allowed us to find impaired ER protein folding, which resulted in a weak UPR when yeast cells were cultured at their maximum growth temperature.

## 2. Results

### 2.1. Development of the Real-Time qPCR-based Method for High-Sensitive Monitoring of HAC1-mRNA Splicing

**Figure 1:**
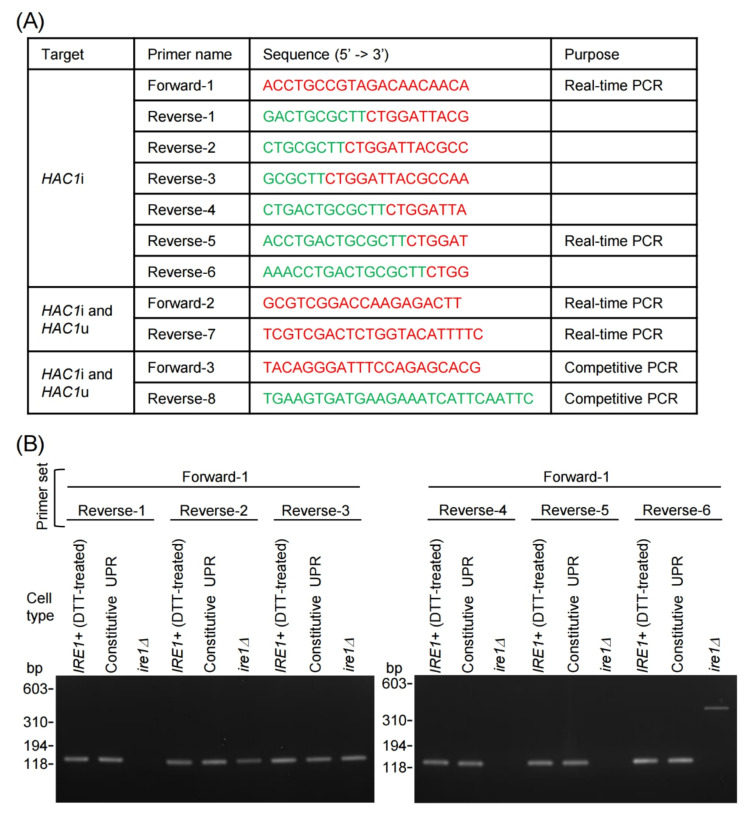
Oligonucleotide design for PCR primers that specifically amplify the *HAC1*i cDNA. (**A**) PCR primers used in this study. The red and green letters represent sequences hybridizing to the first and second HAC1 exon, respectively. (**B**) Through the oligo(dT)-primed RT reaction, total RNA samples obtained from DTT-treated (3 mM, 30 min) *IRE1+* cells (YTH001), unstressed constitutive-UPR cells (YKY1002), and unstressed *ire1Δ* cells (YTH002) were converted to cDNA, which was then used as the template for PCR with the indicated primer sets. The PCR products were run on 2% agarose.

At the beginning of this study, we developed a PCR method to amplify *HAC1*i cDNA, but not *HAC1*u cDNA. While *HAC1*u cDNA contains an intron sequence (blue letters in Supplementary Figure S1), two exon sequences (red and green letters in Supplementary Figure S1) are directly joined in *HAC1*i cDNA. As shown in Figure 1A, we designed reverse PCR primers to hybridize to the exon joint site (Reverse-1 to Reverse-6), which may act as *HAC1*i-specific primers. The forward primer was designed to hybridize to the first exon (Figure 1A; Forward-1).

In the experiment shown in Figure 1B, we checked if, as expected, the primer sets specifically amplified *HAC1*i cDNA. As PCR templates, we used oligo(dT)-primed cDNA generated from total RNA from constitutive-UPR cells, *ire1Δ* cells, and DTT-treated *IRE1+* cells. Since the constitutive-UPR cells possess a mutation that removes the *HAC1*u intron sequence, they constitutively produce *HAC1*i mRNA but not *HAC1*u mRNA [10]. DTT-treated *IRE1+* cells also carried abundant *HAC1*i mRNA. On the other hand, *ire1Δ* cells were unable to perform *HAC1*-mRNA splicing and thus possessed only *HAC1*u mRNA.

Figure 1B shows the PCR product not only from constitutive-UPR cells and the *IRE1+* cells, but also from *ire1Δ* cells using Reverse-2, Reverse-3, or Reverse-6 as the reverse PCR primer. Considering the band size, we speculate that Reverse-2 and Reverse-3 hybridized to the first-exon/intron junction of the *HAC1*u cDNA, while Reverse-6 hybridized to the intron/second-exon junction. Conversely, Reverse-1, Reverse-4, and Reverse-5 produced an expected result when used as the reverse PCR primers. Amplicon bands, the size of which was also as we expected, were only observed when we used the cDNA samples from constitutive-UPR cells or *IRE1+* cells as the PCR template.

**Figure 2:**
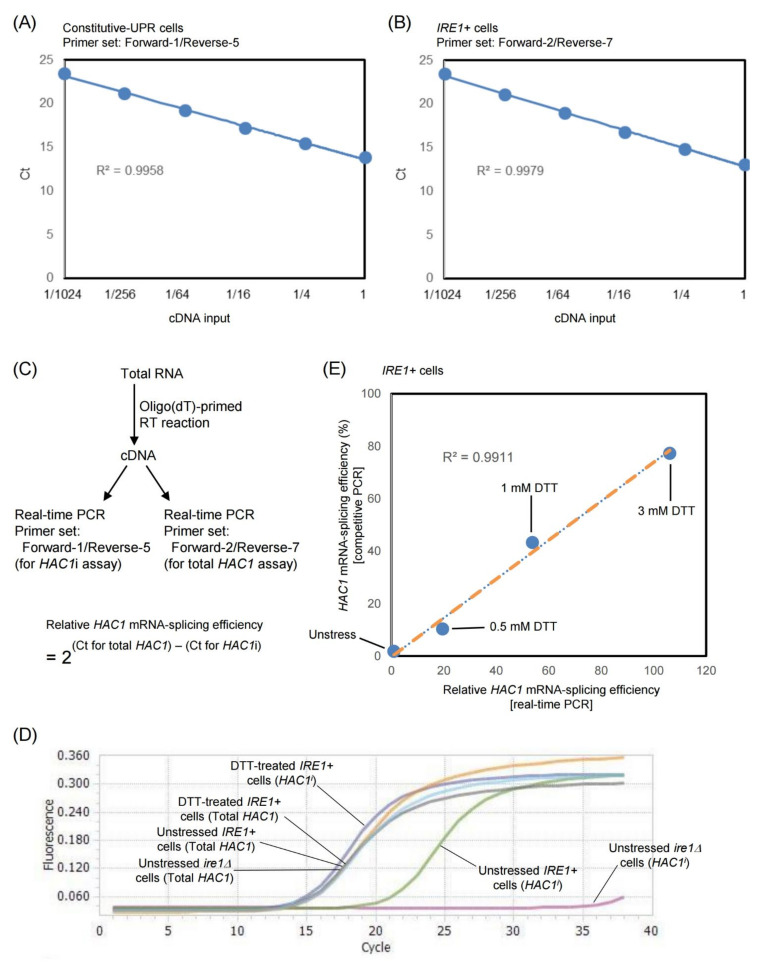
Quantitative measurement of the relative efficiency of *HAC1*-mRNA splicing through real-time PCR. (**A**) The cDNA sample generated by the oligo(dT)-primed RT reaction from the total RNA of constitutive-UPR cells (YKY1002) was 4-fold serially diluted and was subjected to real-time PCR in which the Forward-1/Reverse-5 primer set was used. (**B**) The cDNA sample generated by the oligo(dT)-primed RT reaction from total RNA of *IRE1+* cells (YTH001) was 4-fold serially diluted and subjected to real-time PCR in which the Forward-2/Reverse-7 primer set was used. (**C**) The procedure for the real-time qPCR-based estimation of the relative *HAC1* mRNA-splicing efficiency. (**D**) Amplification curves of the real-time PCR analysis. The oligo(dT)-primed cDNA samples were produced from RNA that was extracted from DTT-treated (3 mM, 30 min), unstressed *IRE1+* cells (YTH001), and unstressed *ire1Δ* cells (YTH002). The primer sets are shown in panel C. (**E**) *IRE1+* cells (YTH001) were stressed by the indicated concentrations of DTT for 30 min and checked for *HAC1*-mRNA splicing with the two different methods. The x-values were normalized against that of unstressed cells, which was set at 1.0.

Thus, we decided to use the primer set Forward-1/Reverse-5 for *HAC1*i-specific PCR and checked if it can be used for real-time quantitative PCR. The oligo(dT)-primed cDNA sample generated from constitutive-UPR cells was 4-fold serially diluted and was subjected to real-time PCR analysis (Figure 2A). As expected, the Ct value and the input cDNA volume correlated linearly. For the normalization control, we used another primer pair, Forward-2/Reverse-7, which hybridizes to *HAC1′*s first exon sequence and thus serves as a primer set to measure total *HAC1* (*HAC1*u + *HAC1*i) abundance. Similar to Forward-1/Reverse-5 (Figure 2A), the Forward-2/Reverse-7 primer set was likely to enable us to perform an efficient and quantitative PCR (Figure 2B). Therefore, we propose that the relative abundance of *HAC1*i mRNA that is normalized against the total *HAC1*-mRNA abundance, namely, the relative *HAC1* mRNA-splicing efficiency, can be calculated through the formula shown in Figure 2C.

When we used the Forward-2/Reverse-7 primer pair to monitor total *HAC1* abundance using real-time RT-qPCR, samples from unstressed *IRE1+* cells, DTT-treated *IRE1+* cells, and unstressed *ire1Δ* cells exhibited similar amplification curves (Figure 2D). On the other hand, no amplification was observed from *ire1Δ* cells when we used the Forward-1/Reverse-5 primer pair, which specifically amplifies the *HAC1*i sequence. In contrast, total RNA from DTT-treated *IRE1+* cells contained a high abundance of *HAC1*i mRNA. Figure 2D also shows that the low-abundance *HAC1*i mRNA in unstressed *IRE1+* cells was clearly detectable using this method.

A traditional and common method for monitoring the *HAC1*-mRNA splicing is to perform competitive PCR under saturating conditions [7]. Upon competitive PCR, *HAC1*i cDNA and the *HAC1*u cDNA yielded different-size PCR products because the forward and reverse primers were designed to hybridize to the first and second exon, respectively. In the experiment shown in Supplementary Figure S2, total RNA samples were converted to cDNA through the oligo(dT)-primed RT reaction, used as PCR templates, and then analyzed on agarose-gel electrophoresis. While the *HAC1*i bands from DTT-treated *IRE1+* cells were clearly visible (Supplementary Figure S2A; lane 3), unlike the case of the real-time qPCR-based method developed in this study, the low-abundance *HAC1*i mRNA in unstressed *IRE1+* cells was hardly detected by conventional competitive PCR (Supplementary Figure S2A; lanes 2). This observation underscores an advantage of our real-time qPCR-based method, which was sensitive enough to quantitatively monitor low-level *HAC1*-mRNA splicing.

To further evaluate the real-time qPCR-based method, we next monitored the *HAC1*-mRNA splicing in *IRE1+* cells treated with different concentrations of DTT (Supplementary Figure S2B and Figure 2E). The strong linearity of the plots shown in Figure 2E indicates that the real-time qPCR-based method serves as an effective alternative for quantitative monitoring of *HAC1*-mRNA splicing.

### 2.2. Weak Induction of the UPR during Cell Growth at Maximum Growth Temperature

**Figure 3:**
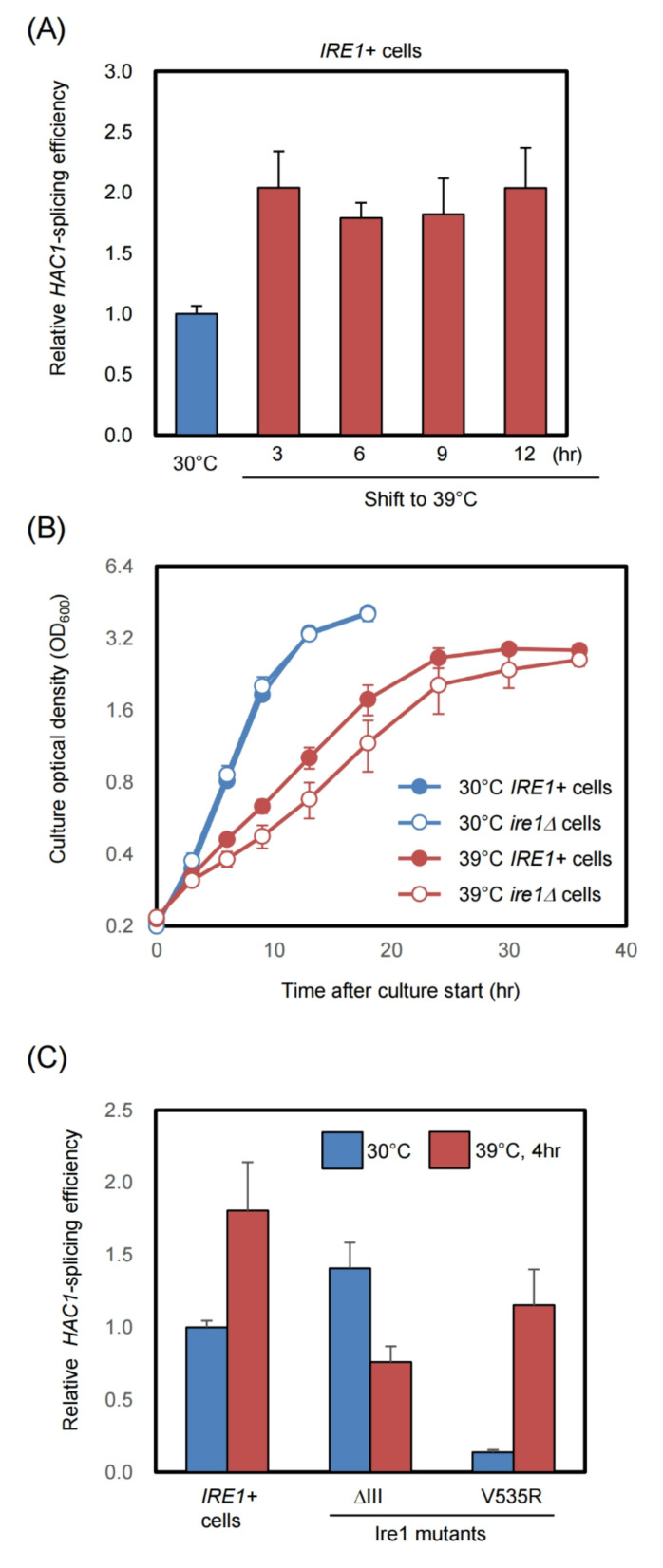
UPR induction in cells grown at the upper-limit growth temperature of 39 °C. (**A**) and (**B**) after preculturing at 30 °C, the *ire1Δ* cells Y11907 transformed with the single-copy IRE1 plasmid pRS313-IRE1 (*IRE1+*) or the control empty vector plasmid pRS313 (*ire1Δ*) were shifted to 39 °C or were continuingly grown at 30 °C for monitoring of the relative *HAC1* mRNA-splicing efficiency and the culture optical density. (**C**) The *ire1Δ* strain Y11907 transformed with pRS313-IRE1 (*IRE1+*) or its mutants (ΔIII or V535R) were incubated and analyzed as shown in panel A. To monitor the relative *HAC1* mRNA-splicing efficiency, cells were harvested at the exponential growth phase, and the experimental procedure shown in Figure 2C was employed. Values in A and C were normalized against that of *IRE1+* cells cultured at 30 °C, which were set at 1.0.

Since our real-time qPCR-based method enables quantitative monitoring of the *HAC1*-mRNA splicing even at low abundance, we used this method to search for conditions that provoke UPR in yeast. Yeast cells used in this study are derived from standard *S. cerevisiae* strains, which grow rapidly at their optimal growth temperature of 30 °C, but proliferate more slowly at their maximum growth temperature of 39 °C. In the experiment shown in Figure 3A, *IRE1+* cells were shifted from 30 °C to 39 °C or were grown at 30 °C without the temperature shift. The temperature shift from 30 °C to 39 °C resulted in an approximately 2-fold increase in the *HAC1* mRNA-splicing efficiency that was maintained for at least 12 h after the temperature shift. The increase in *HAC1*-mRNA splicing shown here could not be observed with the conventional competitive PCR method (Supplementary Figure S2C).

Next, we compared the growth of *IRE1+* and *ire1Δ* cells at different temperatures. At 39 °C, *ire1Δ* cells, which cannot induce *HAC1*-mRNA splicing, grew slower than *IRE1+* cells (Figure 3B). In contrast, *IRE1+* and *ire1Δ* cells grew at almost the same rate at 30 °C. This observation indicates that the weak UPR induction at 39 °C is a physiologically meaningful event that supports cellular growth in this harsh condition.

As described in the introduction, Ire1 is known to be activated by two different types of ER-stressing stimuli via distinct mechanisms. Unfolded proteins accumulated in the ER are directly sensed by the Ire1 luminal domain, which is then self-associated for the UPR induction [5,6]. On the other hand, the transmembrane domain of Ire1 has a unique structure that leads to self-association and activation of Ire1 in response to LBS [7,8]. We previously reported that a partial deletion mutation of the yeast Ire1 luminal domain, which is named the ΔIII mutation, specifically impairs unfolded protein-dependent activation of Ire1 without affecting the UPR evocation by LBS [7,11]. In contrast, a transmembrane-domain point mutation, V535R, of yeast Ire1 specifically impairs the LBS-dependent activation of Ire1 [8,11]. As shown in Figure 3C, the *HAC1*-mRNA splicing in cells carrying the ΔIII mutant version of Ire1, which was slightly higher than that in wild-type *IRE1+* cells at 30 °C, was not boosted, but was rather lowered by the temperature shift from 30 °C to 39 °C. Thus, we deduce that this temperature shift activates Ire1 via impairment of protein folding in the ER. In contrast, the *HAC1*-mRNA splicing in cells carrying V535R Ire1 was considerably lower than that in wild-type *IRE1+* cells at 30 °C, and was strongly increased by the temperature shift (Figure 3C). The minor UPR in wild-type *IRE1+* cells cultured at 30 °C may therefore be due to LBS, which is provoked even under unstressed conditions. However, LBS is unlikely to be involved in the weak induction of UPR due to a high growth temperature.

## 3. Discussion

Ire1 is an ER-stress sensor and an UPR initiator conserved across eukaryotes. This study aimed to discover scenarios that provoke ER stress and activate Ire1 in *S. cerevisiae*. One experimental approach frequently employed for checking cellular UPR level is to monitor the expression of the Hac1-target genes. However, this method is indirect and may result in occasional biases. If *HAC1*i mRNA is poorly translated, Hac1-target gene expression is low, even in ER-stressed cells in which Ire1 is highly activated [12]. In contrast, expression of the most prominent Hac1-target gene, *KAR2*, is also upregulated by heat shock independently of UPR [13], though *KAR2* expression is frequently regarded as an indicator for cellular ER-stress and UPR levels. To monitor Ire1 activation and UPR in yeast, we thus directly checked *HAC1*-mRNA splicing, which is the proximal event provoked by Ire1 activation.

As aforementioned herein, a conventional method to monitor the *HAC1*-mRNA splicing is the competitive PCR analysis of poly(dT)-primed cDNA samples. However, this method is not reliable when the *HAC1* mRNA-splicing level is low. More canonically, *HAC1*-mRNA splicing has been checked by northern blot detection of *HAC1* species. However, this method is also hardly capable of the quantitative detection of low-abundance *HAC1*i mRNA in unstressed or weakly ER-stressed cells. Thus, by using a PCR primer set that specifically and efficiently amplifies the *HAC1*i sequence but not the *HAC1*u sequence, we developed a real-time PCR-based method for the quantitative detection of *HAC1*i mRNA (Figure 2C). Our results in Figure 2 indicate that this new method enables a highly sensitive and quantitative monitoring of the *HAC1*-mRNA splicing. A notable merit of this method is that it allows us to quantitatively detect low-level *HAC1*i.

In animal cells, IRE1α (the major paralogue of Ire1) promotes splicing of the XBP1 mRNA [1,2]. While XBP1-mRNA splicing was initially monitored by competitive PCR, recent studies have developed real-time PCR-based methods to specifically and quantitatively detect the spliced XBP1 form [14,15]. Thus, we believe that the experimental procedures involving real-time PCR will become a standard strategy for monitoring Ire1′s activity in a wide variety of eukaryotes.

Using the method developed herein, we found that *HAC1*-mRNA splicing was weakly induced at the maximum growth temperature in yeast cells (Figure 3A). Due to growth retardation caused by the *ire1Δ* mutation (Figure 3B), we deduced that this phenomenon is important, albeit not essential, for cellular growth under this extreme condition. In other words, weak UPR that is only detectable with our method is physiologically meaningful. We previously reported that in *S. cerevisiae*, the UPR is induced under various conditions that include zinc depletion, diauxic shift, and exposure to cadmium, ethanol, or acetic acid [16,17,18,19,20]. Based on insights gained from these previous reports and the current study, we propose that the UPR is a cellular protective response for coping with a wide variety of environmental changes and stressing stimuli in yeast cells.

High temperatures are known to impair the protein folding status. On the other hand, membrane-lipid fluidity is elevated when cells are cultured at high temperatures, possibly leading to LBS. Our observations in Figure 3C indicate that the weak UPR induced by culturing yeast cells at their maximum growth temperature is likely due to ER accumulation of unfolded proteins, but not to LBS. In other words, it is likely that the UPR contributes to re-folding or removal of ER-accumulated unfolded proteins that are produced upon culturing yeast cells at high temperatures. Plant cells also induce UPR when exposed to high temperatures [21,22]. To the best of our knowledge, our present study is the first report to suggest a possible mechanism for UPR induction at high temperatures.

Our findings shown in Figure 3 also illustrate what happens in unstressed yeast cells cultured at 30 °C. The faint *HAC1*-mRNA splicing that occurs under this condition is probably due to LBS because it was compromised by the V535R mutation in Ire1 (Figure 3C). It is therefore likely that even under normal conditions, yeast cells suffer from weak LBS. This insight is in contrast to that of another yeast species, *Komagataella phaffii* (*Pichia pastoris*), which exhibits strong *HAC1*-mRNA splicing even under unstressed conditions, probably due to high protein load in the ER [23]. Although Bicknell et al. [24] previously reported that growth of *S. cerevisiae* cells was slightly retarded under unstressed conditions, suggesting a housekeeping function of the UPR, this was not observed in this study (Figure 3B). At least in our studies, ER stress in cells cultured under normal conditions did not seem to be strong enough to interfere with cellular growth.

In conclusion, here, we described a real-time PCR-based method to quantitatively monitor low-level UPR in yeast cells, which demonstrated UPR induction by culturing cells at their maximum growth temperature. We anticipate that, in the future, this method will be applicable to the study of other stressing situations that induce weak ER stress and a minimal UPR.

## 4. Materials and Methods

### 4.1. Yeast Strains

The *S. cerevisiae* strain Y11907 (*MATα ura3-Δ0 leu2-Δ0 his3-Δ1 lys2-Δ0 ire1::kanMX4*) is an *IRE1*-deletion derivative of the standard strain BY4742 and was obtained from EUROSCARF (Available online: http://www.euroscarf.de (accessed on 29 January 2022)). To obtain *IRE1+* cells, Y11907 was transformed with the yeast centromeric plasmid pRS313-IRE1 carrying the *IRE1* gene [25]. Its congenic *ire1Δ* derivative was obtained through transformation of Y11907 with an empty vector pRS313 [26]. To generate a constitutive-UPR strain YKY1002, the *HAC1*u intron sequence on the Y11907 genome was deleted in our previous study [10]. A partial deletion mutation (ΔIII) and a point mutation (V535R) were introduced into the *IRE1* gene on pRS313-IRE1, as previously described [13].

Another *IRE1*-deletion strain KMY1015 (*MATα leu2-3,112 ura3-52 his3-Δ200 trp1-Δ901 lys2-801 ire1::TRP1*) [27] was also used. For generation of the *IRE1+* strain YTH001 and the congenic *ire1Δ* strain YTH002, KMY1015 was transformed with pRS313-IRE1 and pRS313, respectively.

### 4.2. Yeast Culturing and Stress Imposition

The standard synthetic dextrose (SD) medium contained 2% glucose, 0.67% yeast nitrogen base without amino acids (Difco), and appropriate auxotrophic requirements. For preculturing, yeast cells were aerobically incubated overnight at 30 °C in SD medium. The precultures were then diluted in SD medium to an OD_600_ of 0.20 and were further shaken aerobically at 30 °C. For culturing at 39 °C, the culturing temperature was shifted from 30 °C to 39 °C just after dilution of the precultures. A UV-1800 spectrophotometer (Shimazu) was used to measure the optical density at 600 nm (OD_600_) of cultures.

DTT (Nacalai Tesque) was dissolved in water to make a 1 M stock solution, which was then added to the cultures at the exponential growth phase.

### 4.3. RNA Analysis

The hot phenol method was used for the extraction of total RNA from yeast cells [28]. For the reverse transcription (RT)-competitive PCR procedure, total RNA samples were subjected to the RT reaction using the oligo(dT) primer, as previously described [7]. The resulting cDNA samples were then used as templates to saturate the PCR amplification of the *HAC1* species. The primers used were 5′-TACAGGGATTTCCAGAGCACG-3′ (Forward-3) and 5′-TGAAGTGATGAAGAAATCATTCAATTC-3′ (Reverse-8) [7,29]. For size-dependent fractionation, the PCR products were electrophoretically run on 2% agarose gels in TBE buffer. DNA bands in the gels were stained with ethidium bromide and were quantitatively detected by the ChemiDoc imaging system (BioRad, Hercules, CA, USA). The *HAC1*-mRNA splicing efficiency was calculated using the following formula:

(*HAC1*i band intensity)/{(*HAC1*u band intensity) + (*HAC1*i band intensity)}

To evaluate primer candidates for *HAC1*i-specific PCR, the same procedure was performed using them.

To convert total RNA to cDNA for real-time quantitative RT-PCR (RT-qPCR) analysis, the oligo(dT)-primed RT reaction was performed using the PrimeScript II Reverse Transcriptase Kit (Takara) as described in the manufacturer’s instruction. The resulting cDNA samples were then analyzed with the real-time PCR machine LightCycler 96 (Roche) using TB Green Premix Ex Taq II (Takara). The PCR temperature protocol was as follows: an initial denaturation at 95 °C for 5 min, followed by amplification cycles at 95 °C for 10 s, 60 °C for 10 s, and 72 °C for 20 s. The primer set for the *HAC1*i-specific amplification was 5′-ACCTGCCGTAGACAACAACA-3′ (Forward-1) and 5′-ACCTGACTGCGCTTCTGGAT-3′ (Reverse-5). The primer set for total-*HAC1* (*HAC1*i and *HAC1*u) amplification was 5′-GCGTCGGACCAAGAGACTT-3′ (Forward-2) and 5′-TCGTCGACTCTGGTACATTTTC-3′ (Reverse-7). The relative abundance of *HAC1*i against total *HAC1* was calculated using the following formula:

2^(Ct for total *HAC1*) − (Ct for *HAC1*i)^

### 4.4. Statistics

The relative *HAC1* mRNA-splicing efficiency and cellular growth at different culturing temperatures are presented as the averages and the standard deviations from triplicate biological replicates in which independent transformants of Y11907 were employed.

## Data Availability

The data underlying this article will be shared on reasonable request to the corresponding author. All data are officially arcived in the Research Data Preservation System managed by Nara Inst. Sci. Tech.

## Supplementary Materials

The following supporting information can be downloaded at: https://www.mdpi.com/article/10.3390/ijms23031669/s1.
