# Supplementary material for: Induction of the Unfolded Protein Response at High Temperature in Saccharomyces cerevisiae
Source: Int J Mol Sci. 2022 Jan 31;23(3):1669. doi: 10.3390/ijms23031669 (PMC8836091; doi:10.3390/ijms23031669)
Supplement: Supplementary file 1 [file ijms-23-01669-s001.zip › Supplemental/Supplemental-Compressed.pdf]

Supplemental Figure S1

First Exon: Red
Intron: Blue
Second Exon: Green

```
5'— ATGGAAATGACTGATTTTGA······CGCCCAAGAGTATGCGCGATTCCGCGTCGGACCAAGAGACTTCATGGGAGCTG
3'— TACCTTTACTGACTAAAACT······GCGGGTTCTCATACGCGCTAAGGCGCAGCCAGGTTCTCTGAAGTACCCTCGAC

    CAGATGTTTAAGACGGAAAATGTACCAGAGTCGACGACGCTACCTGCCGTAGACAACAACAATTTGTTTGATGCGGTG
    GTCTACAAATTCTGCCTTTTACATGGTCTCAGCTGCTGCGATGGACGGCATCTGTTGTTGTTAAACAAACTACGCCAC

    GCCTCGCCGTTGGCAGACCCACTCTGCGACGATATAGCGGGAAACAGTCTACCCTTTGACAATTCAATTGATCTTGACA
    CGGAGCGGCAACCGTCTGGGTGAGACGCTGCTATATCGCCCTTTGTCAGATGGGAAACTGTTAAGTTAACTAGAACTGT

    ATTGGCGTAATCCAGCCGTGATTACGATGACCAGGAAACTACAGTGAACAAGAACACTAGCCCCAGCTTTTGCTTTCTG
    TAACCGCATTAGGTCGGCACTAATGCTACTGGTCCTTTGATGTCACTTGTTCTTGTGATCGGGGTCGAAAACGAAAGAC

    CTTTTTTTCTTTTTTTTTTTTTTTAGTCGTGGTTCTCTGATGGGGGAGGAGCCGGTTAAAGTACCTTCAAAAGCAGAATG
    GAAAAAAAGAAAAAAAAAAAAAAATCAGCACCAAGAGACTACCCCCTCCTCGGCCAATTTCATGGAAGTTTTCGTCTTAC

    CAGGGTTATTGGAAGCTTTCTTTTTTTCTTTTATGCTAGTTTTTCCTGAACAAATAGAGCCATTCTTTTCTTATTACTAAG
    GTCCCAATAACCTTCGAAAGAAAAAAAGAAAATACGATCAAAAAGGACTTGTTTATCTCGGTAAGAAAAGAATAATGATTC

    AAATGGACGGCTTGCTTGTACTGTCCGAAGCGCAGTCAGGTTTGAATTCATTTGAATTGAATGATTTCTTCATCACTTCATGA —3'
    TTTACCTGCCGAACGAACATGACAGGCTTCGCGTCAGTCCAAACTTAAGTAAACTTAACTTACTAAAGAAGTAGTGAAGTACT —5'
```

Supplemental Figure S1. Sequence of the *HAC1* gene

The red, blue, and green letters represent nucleotide sequences of the first exon, the intron, and the second exon, respectively.

Supplemental Figure S2

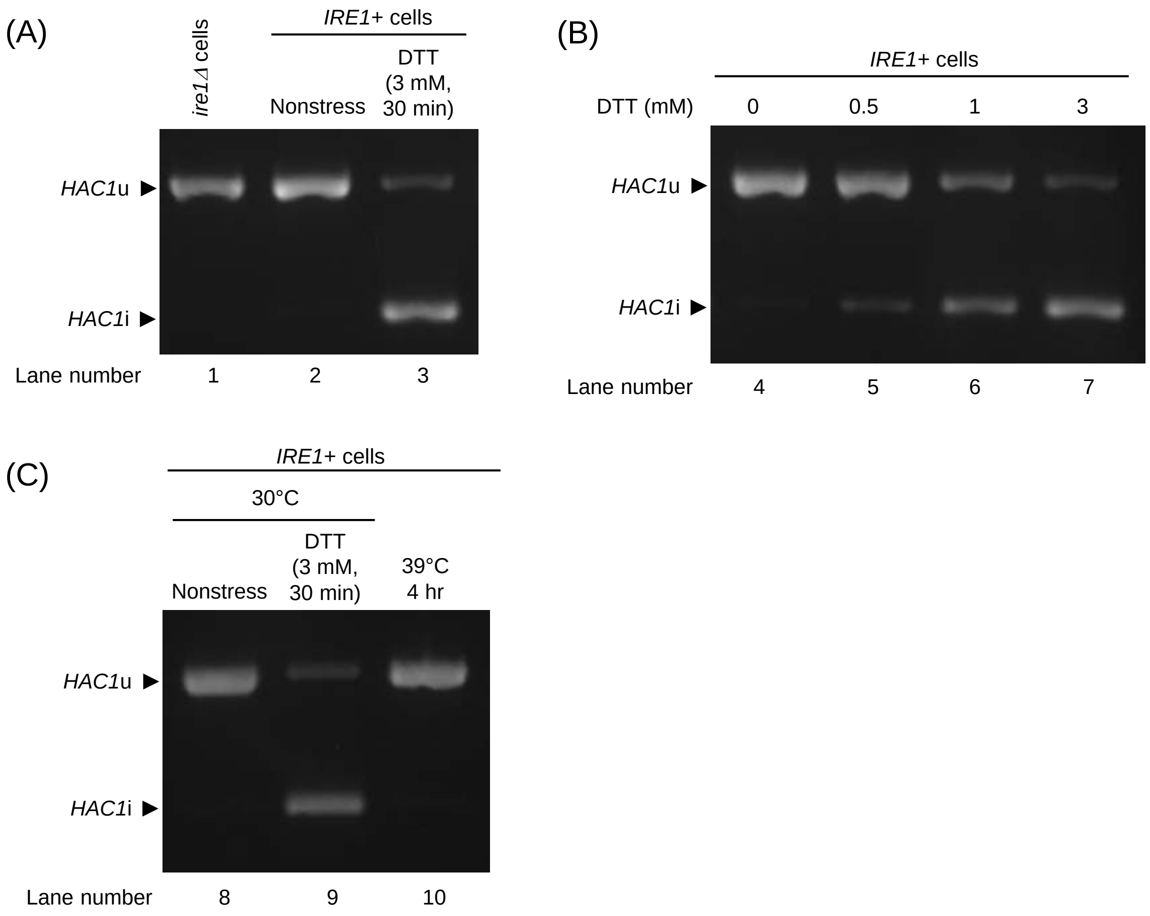

Supplemental Figure S2. The raw electrophoresis-gel image of the competitive PCR-based estimation of the *HAC1*-mRNA splicing

After being converted to cDNA by the oligo(dT)-primed RT reaction, total RNA samples were subjected to competitive PCR analysis. (A) RNA samples analyzed in Fig. 2D. (B) RNA samples analyzed in Fig. 2E. (C) The *ire1Δ* strain Y11907 transformed with the single-copy *IRE1* plasmid pRS313-IRE1 (*IRE1+*) was stressed as indicated before the total RNA extraction.
