# Supplementary figures and images for: Induction of the Unfolded Protein Response at High Temperature in Saccharomyces cerevisiae
Source: Int J Mol Sci. 2022 Jan 31;23(3):1669. doi: 10.3390/ijms23031669 (PMC8836091; doi:10.3390/ijms23031669)

Figure 1B

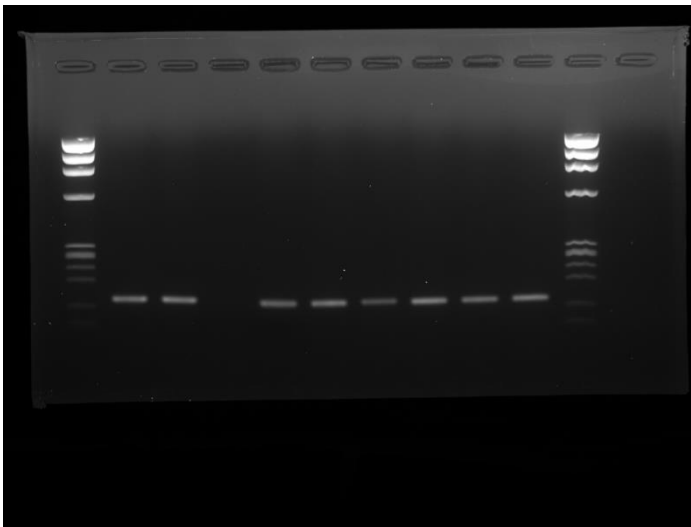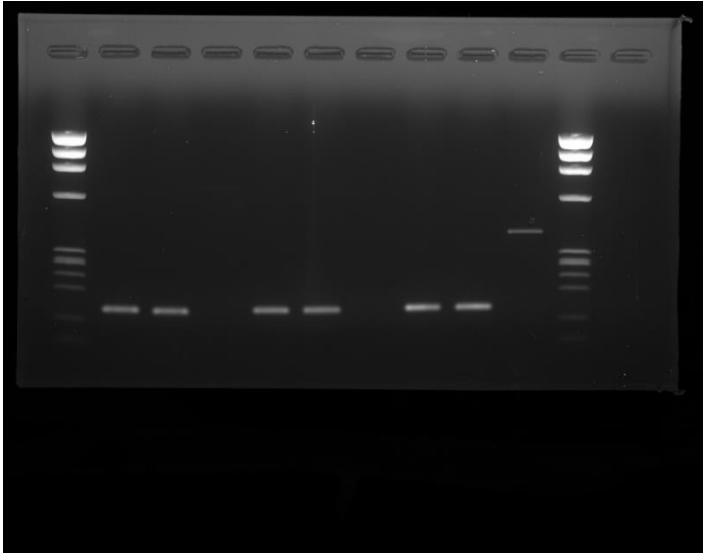

Figure S2A &B

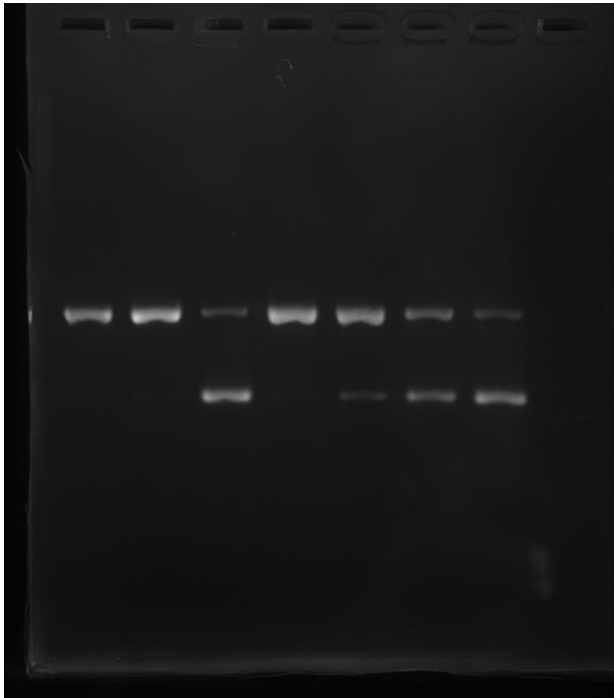

Figure S2C

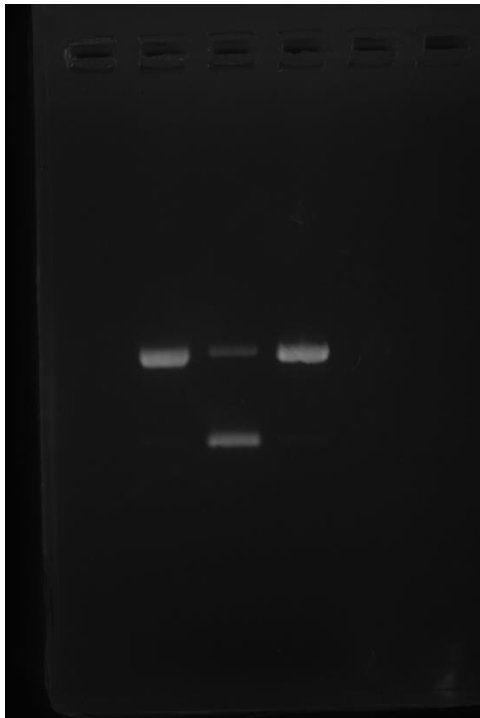

Supplement: Supplementary file 1 [file ijms-23-01669-s001.zip › Supplemental/Original Gel.pdf]
